# A facility level cross-sectional survey to assess health system readiness for oral cancer care in Madhya Pradesh, India

**DOI:** 10.6026/973206300214092

**Published:** 2025-11-15

**Authors:** Sana Anwar, Anindo Majumdar, Yogesh Damodar Sabde, Pankaj Goel, Sanjeev Kumar, Ujjawal Khurana

**Affiliations:** 1 Department of Community and Family Medicine, All India Institute of Medical Sciences, Bhopal, Madhya Pradesh, India; 2 Scientist F and Head, Environmental Health and Epidemiology, ICMR-- National Institute for Research in Environmental Health (ICMR - NIERH), Bhopal, Madhya Pradesh, India; 3 Faculty of Medical Research, Academy of Scientific and Innovative Research (AcSIR), Ghaziabad, Uttar Pradesh, India; 4 Department of Dentistry, All India Institute of Medical Sciences, Bhopal, Madhya Pradesh, India; 5 Department of Pathology and Lab Medicine, All India Institute of Medical Sciences, Bhopal, Madhya Pradesh, India

**Keywords:** Health system, India, oral cancer

## Abstract

Oral cancer is included under national NCD programs in India due to its high burden, societal and economic impact. Therefore, it is of interest to assess the readiness of government healthcare facilities in two districts of Madhya to deliver good quality oral cancer care. Assessment of 58 facilities revealed that opportunistic screening and telemedicine was functional and availability of manpower and supportive drugs was good. Shortcomings in availability of diagnostics, cancer medicines, information, education and communication (IEC) activities, dentists, dieticians and counsellors were found. Further strengthening of oral cancer services under the NCD programs is essential for comprehensive oral cancer care.

## Background:

The National Programme for Prevention and Control of non-communicable diseases (NP-NCD) being implemented by Government of India addresses the growing burden of non-communicable diseases [1]. A major component of this programme is the early detection and screening of common cancers i.e., oral, breast and cervical for individuals aged 30 years and above at all public healthcare facilities through both universal screening and opportunistic screening [2]. Oral cancer has been included under NP-NCD due its high prevalence in India, where the disease accounts for nearly one third of the global burden and is strongly associated with widespread tobacco and areca nut consumption [3]. Oral visual inspection for oral cavity lesions, tobacco cessation services, information, education and communication (IEC) activities, use of NCD mobile application, telemedicine, training and timely referral mechanisms related to oral cancer prevention, care and control are also emphasized under this program [4]. Despite these efforts, program implementation and coverage of oral cancer screening remains low and varies significantly across different states and districts, depending on local leadership, infrastructure, manpower availability, logistics and community participation [5]. According to National Family Health Survey-5, only a small proportion of eligible adults, i.e. less than 10% in many states reports ever being screened for oral cancer [6]. Health system preparedness for oral cancer screening has been studied in India and other low and middle income countries (LMIC) and the findings highlighted the need for structured training, supportive supervision, financing and integration with primary care for ensuring program success [7, 8]. Globally, health system analyses from LMICs emphasize many challenges where studies from rural South Asia and Africa point to the absence of decentralized diagnostic services, dependence on tertiary centres and irregular supply of essential medicines as major obstacles in cancer control efforts [9]. This holds true for Indian context too, where many primary and secondary health centres still lack diagnostic facilities, forcing patients to travel to distant tertiary hospitals and so, it is crucial to document the service delivery, manpower availability and logistics including diagnostic investigations, medicine stocks and infrastructure of the primary and secondary level healthcare facilities as this would help to understand how ready their health systems are for oral cancer related services and what are the gaps that needs to be addressed [10]. Therefore, it is of interest to report the availability and functionality of oral cancer related services at different levels of government healthcare facilities namely District Hospitals (DH), Community Health Centres (CHC), Primary Health Centres (PHC) and Ayushman Arogya Mandirs (AAMs).

## Methodology:

The present study was a cross-sectional facility-based survey conducted in Bhopal and Raisen districts of Madhya Pradesh, India from October to December 2023. The list of all the eligible facilities as per the NP-NCD guidelines i.e., DHs, CHCs, PHCs and AAMs was collected from the office of the respective district authorities after due permissions. Bhopal (District 1 in the current study), is primarily an urban district and has one DH, two CHCs, eight urban PHCs (UPHCs), 10 PHCs (rural) and 71 AAMs. Raisen (District 2 in the current study), is primarily a rural district and has one DH, eight CHCs, 18 PHCs (rural) and 161 AAMS. In both the districts, each PHC (rural) had AAMs under them, which are the Health and wellness centre- sub centres (HWC-SCs). One AAM (nearest to PHC) was included in the study conveniently out of all the AAMs under that PHC. Data collection was done by the investigator (SA) by physically travelling to the selected facilities and was started after obtaining ethical approval from the Institutional Human Ethics Committee (IHEC-SR/PhD/July/22/01). As the present survey was the part of umbrella implementation research, which also consists of a community-based cluster randomized trial, Clinical Trial Registry-India (CTRI) registration was also done (Reg. no. CTRI/2023/11/060380). Informant for facility related data were mostly administrative in-charge of the respective facilities. Written informed consent was obtained from all the participants prior to the data collection. A facility level survey checklist was prepared based on the NP-NCD monitoring framework and operational guidelines and this was supplemented by using standards recommended by the Indian Public Health Standards (IPHS 2022), wherever required [11]. Some variables which were not as per NP-NCD and IPHS 2022 guidelines, but were important in the context of oral cancer care for capturing any specific initiatives by the particular healthcare facility, were also included, such as availability of dedicated hospital beds for cancer related admissions and day care chemotherapy unit for patients with oral cancer. The checklist covered variables such as basic infrastructure i.e, internet connectivity, electricity connection and ambulance availability, service delivery i.e, function of NCD clinics, screening of oral cancer, diagnosis and management, Comprehensive Primary Health Care Non-Communicable Disease app (CPHC NCD app) access, availability of IEC materials, telemedicine and mHealth related services, oral cancer related referral services, positive case reporting, sanction of training at the facilities, manpower availability (during last 3 months), etc. Manpower availability was looked at in terms of the availability of specialist doctors, medical officers, nurses, technicians, pharmacist, ASHAs, CHOs and other supporting staff. Availability of medicines and diagnostics (during last 15 days), support systems like palliative care, rehabilitation services, availability of reporting formats, functioning of health management information system (HMIS), financial aid received, as well as organisation of health promotion activities (such as camps) were also captured. Information was gathered face-to-face and to the best of our ability we tried, to verify it by cross checking the information gathered verbally with the registers/relevant documents maintained at the centres/hospitals, wherever feasible, permitted and available. An electronic version of the checklist was developed using Kobo toolbox and data was entered into it individually for each facility by SA. Microsoft excel sheets were extracted from Kobo toolbox and analysis was conducted in terms of availability (Yes/No), reflecting the number of each item of checklist against the standard guidelines mentioned above.

## Results:

A total of 69 government healthcare facilities were eligible to be surveyed as per the list of healthcare facilities obtained from the concerned district authorities. During the survey, seven PHCs in two districts combined were found to be currently not functional due to reasons not known. Also, four CHCs in the district 2 had been recently upgraded to civil hospitals and therefore were excluded from the analysis. The final sample thus comprised 58 facilities- two DHs (one in each district), six CHCs (two in District 1 and four in District 2), 29 PHCs (eight urban PHCs in district 1, nine rural PHCs in district 1 and 12 rural PHCs in district 2) and 21 AAMs (nine in district 1 and 12 in district 2). There were no designated urban PHC in district 2. Overall, opportunistic screening for oral cancer was being carried out in 41 out of 58 healthcare facilities, but the extent and regularity of screening varied (Table 1 - see PDF). Centralized ambulance services (108 toll-free emergency helpline services) were generally available at the PHCs and AAMs. However, no dedicated ambulances were stationed full time at these centres (Table 2 - see PDF). Diagnostic confirmation tests such as biopsy and histopathology were not functional at the district hospitals during the survey (Table 3 - see PDF). All the suspected cases of oral cancer were referred to tertiary care centres. Dedicated beds for admissions and day care chemotherapy unit for oral cancer patients were not available, although this is not a requirement as per NP-NCD guidelines and IPHS 2022 standards. Health promotion and awareness activities were reported from most facilities and were mostly organised in group settings on specific health days. Availability of IEC materials on oral cancer was suboptimal, with posters (in the local language) being the most commonly available material at the centres. Other formats such as videos, pamphlets and booklets were mostly not available at the facilities, except for some facilities. Telemedicine services and the use of CPHC NCD app were more consistently found across the centres. Both doctor-to-patient and doctor-to-healthcare worker interaction through audio and video mode was functional for common NCDs. However, this service was not being utilized for oral cancer related services in most of the facilities. Training on oral cancer was provided at district hospitals but these centres were irregularly utilized as training venues. Supportive drugs for managing the symptoms and complications related to oral cancer such as analgesics, anti-emetics, steroids and antacids were fairly stocked across all the facility levels. Equipment for basic oral examination such as torch, tongue depressor and face mirror were available at higher level facilities but some items were unavailable at the PHCs and AAMs (Table 3 - see PDF). The availability of manpower according to our study was fairly good with specialist physicians, medical officers, nurses, pharmacists, CHOs and ASHAs being available at the appropriate levels of care (Table 4 - see PDF).

## Discussion:

The present study was conducted in 58 public healthcare facilities of Bhopal and Raisen districts of Madhya Pradesh, India and was aimed to assess the health system preparedness for oral cancer service delivery under the public health system. The findings of this study revealed that while facility based opportunistic screening for oral cancer was being conducted in nearly all DH and AAMs and was relatively lower in CHCs and PHCs. This was consistent with the findings of the national tribal preparedness survey done by Kaur *et al.* which showed that only 6% of PHCs conducted oral cancer screening [12]. Our study also showed unavailability of confirmatory diagnostic services such as biopsy and histopathology at DH and CHC levels in the last 15 days. This thus reduces the service delivery to mostly screening and referral, thus increasing the burden on tertiary care centres. The public tertiary care centres are already overburdened and private tertiary care increases the out of the pocket expenditure of patients significantly. Study by Goyal S *et al.* highlighted how reliance on tertiary centres significantly increases financial burden on households [13]. The availability of manpower according to our study was fairly good with physicians, medical officers, nurses, pharmacists, CHOs and ASHAs being available at the appropriate levels of care. But non availability of dentists in more than half of the CHCs, of counsellors/health educator and dieticians at both DHs and CHCs and of physiotherapists at CHC level is a matter of concern, as they are essential for supportive, preventive, clinical and palliative oral cancer care. This finding is in line with the study conducted by Panda *et al.* on health system preparedness of NCDs that highlighted that human resource was inadequate, over-burdened and required specific skills [14]. Shortage/lack of adequate manpower who have specialized roles adversely affects the service delivery mechanism. In our study, although computers and internet connections were available at most facilities, electronic health records (EHR) and health management information systems (HMIS) were absent. Instead, reporting was paper based and was done manually with monthly submissions to the district NCD cell. Madhya Pradesh state monitoring and assessment on real time (SMART) portal is currently active for national oral health program, national program for healthcare of the elderly, national rabies control program and national mental health program but not for NP-NCD [15]. The CPHC-NCD portal which is used by healthcare workers to identify and manage NCDs is neither an EHR nor can be called as a proper HMIS. This gap in infrastructure limits service delivery. This finding aligns with the study done by Krishnan *et al.* also [16]. One of the important findings of our study was the widespread use of the CPHC NCD app for patient entry and use of standard formats (form 4, 3, 2 and 1 for DH, CHC, PHC and AAM respectively) for recording and reporting of oral cancer cases to the district NCD cell. Our study documented fairly good delivery of telemedicine services through e-Sanjeevani platform [17], particularly in urban setting for other diseases but not for oral cancer. Digital technology and innovations can help bridge the gaps in specialist services for remote locations via specialist consultations from higher centres. Pramesh *et al.* in their study reported that during COVID-19 pandemic, tele-consultation could be a good approach for cancer related counselling and follow up in India, especially in rural areas [18]. However, we found telemedicine services being mostly delivered at urban health facilities, this finding may be attributed to better digital infrastructure, specialist availability and internet connectivity in urban areas as compared to rural areas, where network is a common limitation. In present study, chemotherapy drugs recommended under the NP-NCD program were not found even in district hospitals. We could assess the availability of five chemotherapy drugs out of 12. The reason being non-availability of new operational guidelines 2023 for NP-NCD in public domain at the time of protocol finalization, questionnaire development and data collection process. There was optimum availability of supportive drugs such as analgesics, steroids and anti-emetics at all levels of care. This finding was similar to the study done by Ashigbie *et al.* which showed that the availability of medicines increased with increasing level of care of facilities [19]. Ensuring availability of oral cancer specific drugs s per NP-NCD guidelines at secondary care level such as district hospitals could make a substantial difference in the treatment of oral cancer patients. We found that, health promotion activities and awareness programs at the facilities were being conducted mostly in group settings on special occasions such as World No Tobacco Day or Cancer Awareness Weeks. Dependency mostly on posters for health education was also found which might be inadequate, keeping in mind the varying literacy levels and needs of the population. Use of other methods of IEC need to be utilize for better IEC services. Similar gaps in IEC implementation have been reported by Shruti *et al.* where IEC activities often remain irregular and underfunded [20]. The present study also documented the shortage of training sessions for healthcare workers at their workplaces. Although, some induction training was reported, refresher training was missing. This finding is important as this could be a proxy indicator for infrastructure for training at the facilities and on the job training at the facilities. Earlier studies had demonstrated that training healthcare providers can significantly improve early detection rates of oral cancer. Thampi *et al.* showed that community health workers, after appropriate training and supervision, achieved high sensitivity and specificity in detecting suspicious oral lesions, highlighting the potential of task shifting strategies in resource limited settings [21]. One of the strengths of our study was the use of a structured checklist based on both NP-NCD and IPHS guidelines, ensuring a systematic and comprehensive assessment of the health system readiness across all levels of care of the public health system. Data on other useful drugs required for supportive management of oral cancer cases was also documented along with other variables such as availability of dedicated beds for oral cancer related admissions and day care chemotherapy unit for oral cancer patients which enhanced the comprehensiveness of the study. Since the data was collected directly from facilities by physical visits and information was cross checked with registers and documents, the reliability of the findings can be labelled as good. Our study also has some inherent limitations. Availability of certain specific cancer drugs prescribed in the guidelines could not be recorded at the time of visits due to reasons mentioned above. Also, we could not assess all the AAMs of the two districts due to feasibility issues, which could affect the generalizability of the findings to some extent. Equipping DHs and CHCs with diagnostic facilities such as biopsy and histopathology, ensuring the availability of essential cancer medicines at district hospitals alongside supportive drugs at all levels, conducting more trainings at the facilities for all cadres of healthcare workers, expanding routine IEC and community awareness apart from special health days and increasing telemedicine services for oral cancer consultations, particularly in rural areas, through improved digital infrastructure is the need of the hour.

## Conclusion:

This cross-sectional facility-based study across two districts of Madhya Pradesh assessed the implementation of opportunistic oral cancer screening under the NP-NCD program. While screening and referral systems were operational, confirmatory diagnostic services, such as biopsy and histopathology, were not available even at district hospitals. Although basic infrastructure and supportive medicines were fairly good, but gaps in the availability of cancer specific drugs, specialized manpower like dentists, counsellors and dieticians, along with limited IEC activities were noted. Telemedicine services were underutilized for oral cancer care, despite being more common for other diseases in urban areas. Thus, we show that while the foundation for oral cancer services is functional at various levels of care, strengthening efforts are needed to bridge above existing gaps for comprehensive service delivery.

## Financial support and sponsorship: 

Nil
